# Short-term high glucose exposure impairs insulin signaling in endothelial cells

**DOI:** 10.1186/s12933-015-0278-0

**Published:** 2015-08-22

**Authors:** Valeria De Nigris, Gemma Pujadas, Lucia La Sala, Roberto Testa, Stefano Genovese, Antonio Ceriello

**Affiliations:** Insititut d’Investigacions Biomèdiques August Pi i Sunyer (IDIBAPS) and Centro de Investigación Biomédica en Red de Diabetes y Enfermedades Metabólicas Asociadas (CIBERDEM), Hospital Clinic, C/Rosselló, 149-153, 08036 Barcelona, Spain; Experimental Models in Clinical Pathology, INRCA-IRCCS National Institute, Ancona, Italy; Department of Cardiovascular and Metabolic Diseases, IRCCS Gruppo Multimedica, Sesto San Giovanni, MI Italy

**Keywords:** Physiological insulin, High glucose, eNOS, Endothelium, HUVECs

## Abstract

**Background:**

Hyperglycemia is the hallmark of diabetes and its cardiovascular complications. Insulin plays an important role in the regulation of vascular homeostasis and maintenance of endothelial function. Insulin signaling occurs after binding to the insulin receptor, causing activation of two separate and parallel pathways: PI3K/AKT/eNOS and Ras/Raf/MAPK pathways. AKT phosphorylates eNOS at Ser1177, resulting in increased nitric oxide production and vasodilation. The MAPK pathway results in endothelin-1 production and vasoconstriction and mitogenic effects.

**Methods:**

We studied the effects of physiological insulin treatment in human umbilical vein endothelial cells (HUVECs) on the two pathways under high glucose conditions, which mimic the in vivo condition of hyperglycemia. HUVECs were incubated with insulin at different physiological concentrations (from 10^−10^ to 10^−8^ M) for 30 min after 24 h of exposition to normal (5 mmol/L, NG) or high glucose (25 mmol/L, HG). Phosphorylated forms of AKT, eNOS, ERK1/2, p38, JNK and insulin receptor-β subunit (IRβ) were evaluated.

**Results:**

In normal glucose, the active phosphorylated forms of AKT, eNOS, ERK1/2, p38 and JNK were increased in insulin treated cells, in a dose-dependent manner. In high glucose, insulin was not able to activate the PI3K/AKT/eNOS pathway, with the phosphorylated form of eNOS reduced with respect to the control. However, insulin was able to induce the up-regulation of phospho-ERK1/2, -p38 and -JNK. Moreover, we found reduced levels of IRβ phosphorylated form in high glucose as compared to the control. Insulin was able to increase phospho-IRβ in normal glucose but not in high glucose, in which the total protein levels remained reduced.

**Conclusions:**

Exposure to short-term high glucose negatively affects insulin signaling even when physiological insulin concentrations are added. The impairment of the PI3K/AKT/eNOS pathway after physiological insulin treatment could contribute to detrimental effects on cardiovascular homeostasis under high glucose conditions, and might shift toward the activation of certain mitogenic effectors, such as ERK1/2, p38 and JNK, the only ones that respond to physiological insulin treatment in high glucose.

## Background

The pathogenesis of cardiovascular complications in diabetes has been largely described and begins with endothelial dysfunction [1]. The endothelium plays a crucial role in maintaining cardiovascular homeostasis under physiological conditions by releasing a number of vasoactive substances, vasodilators and vasoconstrictors, in balance. They act together to regulate vascular tone, antiproliferation and antiaggregation, and, in addition, to limit increases in blood pressure, to control tissue blood flow and inflammatory responses [2, 3]. The endothelium releases these vasoactive substances in response to mechanical stimuli, such as pressure and shear stress, as well as various hormonal stimuli, such as insulin.

Normal insulin signaling results in glucose uptake by skeletal muscle and fat, suppression of hepatic gluconeogenesis and vasodilation for increased eNOS enzymatic activity in the endothelium [4]. These effects are mediated by insulin binding to the insulin receptor (IR), a ligand-activated tyrosine kinase receptor. Binding and activation of IR results in tyrosine phosphorylation of insulin receptor substrates (IRS) and Shc, leading to the activation of two parallel pathways: the PI3K/AKT/eNOS pathway, also known as the “metabolic arm”, and the Ras/Raf/MAPK pathway, or the “mitogenic/pro-atherogenic arm”. Phosphorylation of IRS-1 leads to the activation of the PI3K/AKT/eNOS pathway. AKT kinase phosphorylates eNOS at Ser1177 and activates it, leading to the production of nitric oxide (NO). On the other hand, phosphorylation of Shc leads to activation of the Ras/Raf/MAPK pathway, resulting in increased endothelin-1 (ET-1) expression and mitogenic effects [5, 6].

Insulin resistance is an important feature of diabetes, obesity, glucose intolerance and dyslipidemia, and it is also a prominent component of cardiovascular disorders, including hypertension, coronary artery disease, and atherosclerosis [7]. In the endothelium, a selective resistance to insulin is observed in the pathological state of hyperinsulinemia [8, 9], when the PI3K/AKT/eNOS pathway is altered, resulting in diminished eNOS activity, reduced NO generation and diminished insulin-mediated vasodilation; by contrast, the Ras/Raf/MAPK pathway is generally preserved, thus ET-1 production and mitogenic effects persist contributing to the vascular effects of insulin resistance [10]. Several studies have investigated the consequences of high insulin levels in vitro and in vivo. In vitro, in human coronary artery endothelial cells (HCAECs) cultured in the presence of 20 mmol/L glucose and treated with an insulin concentration of 50 nmol/L for 10 min, a reduction in insulin-stimulated phosphorylation of eNOS at Ser1177 was observed [11]. In vivo, Kubota et al. [12] demonstrated that the expression levels of insulin-stimulated phosphorylations of AKT and eNOS were decreased by 70–80 % in endothelial cells extracted from the abdominal aorta of 8-week high-fat diet-fed mice and ob/ob mice, treated with an insulin concentration of 100 nmol/L for 30 min, indicating that insulin signaling was impaired in the endothelial cells of these obesity models. While several reports have investigated the role of hyperinsulinemia in the endothelium [11–13] and in the liver [14] and muscle [14, 15], studies in which insulin is used at physiological concentrations are lacking.

The present study aimed to investigate the role of insulin at physiological levels on vascular endothelium in the presence of high glucose concentrations. We hypothesized that the impairment of the insulin signaling may occur in endothelial cells exposed to high glucose, not only at high insulin levels but also at physiological insulin concentrations. In this way, we could help clarify the contribution of insulin to endothelial phenotypes altered by high glucose.

## Methods

### Materials

d-Glucose and insulin were purchased from Sigma-Aldrich (St. Louis, MO, USA).

### Cell culture

HUVECs were purchased from Lonza and cultured in an endothelial cell growth medium-2 (EBM-2) (Lonza Bioresearch LBS, Basel, Switzerland) supplemented with hEGF, FBS, hFGF, heparin, hydrocortisone and GA-1000. It contained less than 10^−12^ M insulin, which was considered to have no effect on our outcome [16]. Cells were harvested at subconfluence and seeded into six-well plates.

### Experimental design

The effects of various concentrations of insulin were examined in HUVECs cultured under normal glucose (5 mmol/L, NG) and high glucose conditions (25 mmol/L, HG) for 24 h. In the last 30 min, HUVECs were treated with increasing physiological concentrations of insulin: 10^−10^ M = 100 pmol/L, 10^−9^ M = 10 nmol/L and 10^−8^ M = 1 nmol/L. After insulin treatment, cells were collected for protein extraction and Western Blot analysis.

### Protein extraction

Cells were harvested and whole cell lysates were prepared using a RIPA buffer (Sigma-Aldrich, St. Louis, MO, USA) with the addition of a protease and phosphatase inhibitor cocktail. Protein content of the lysates was determined using the Bradford reagent (Sigma-Aldrich, St. Louis, MO, USA).

### Western blot analysis

Protein lysates (25 ug) were resolved by SDS–polyacrylamide gel electrophoresis (PAGE-R Gold gels 4–20 %, purchased from Lonza) and transferred to a polyvinylidene fluoride (PVDF) membrane. After blocking with 5 % non-fat dry milk (NFDM) in 20 mM Tris–HCl (pH 7.5), 135 mM NaCl and 0.1 % Tween-20, blots were incubated with monoclonal antibodies against human phospho-AKT (Ser473), phospho-eNOS (Ser1177), phospho-ERK1/2 (Thr202/Tyr204), phospho-p38 (Thr180/Tyr182), phospho-JNK (Thr183/Tyr185), AKT, eNOS, ERK1/2, p38 and JNK (Cell Signaling Technology, Beverly, MA, USA), IRβ (Santa Cruz Biotechnology, CA, USA) and phosphotyrosine (phospho-Tyr) (UBI, Upstate, Lake Placid, NY, USA) (1:1000). Human β-actin (1:1000) (Sigma-Aldrich, St. Louis, MO, USA) was used as a loaded control. Detection was performed using a secondary peroxidase-linked anti-mouse/rabbit antibody (1:3000) (GE Healthcare Europe GmbH, Barcelona, Spain) and an enhanced chemiluminescence system (Pierce Chemical Co, Rockford, IL, USA) according to the manufacturer’s instructions. Proteins were revealed in a CCD camera (ImageQuantLAS4000, GE Healthcare, UK). Protein content quantification was performed using computer-assisted densitometry (http://www.imagej.nih.gov, ImageJ Software, NIH).

## Results

### Acute high glucose enhanced AKT phosphorylation and attenuated eNOS phosphorylation in HUVECs

PI3K/AKT/eNOS is one of the two parallel pathways activated buy insulin after binding to its receptor [5]. Particularly, AKT is an important signaling molecule that is involved in different endothelial functions, for example the regulation of angiogenesis, proliferation, vascular permeability, survival and cellular transformation as well [17]. Also AKT may phosphorylate eNOS to promote vasodilation with increased NO production in endothelial cells [5].

**Fig. 1:**
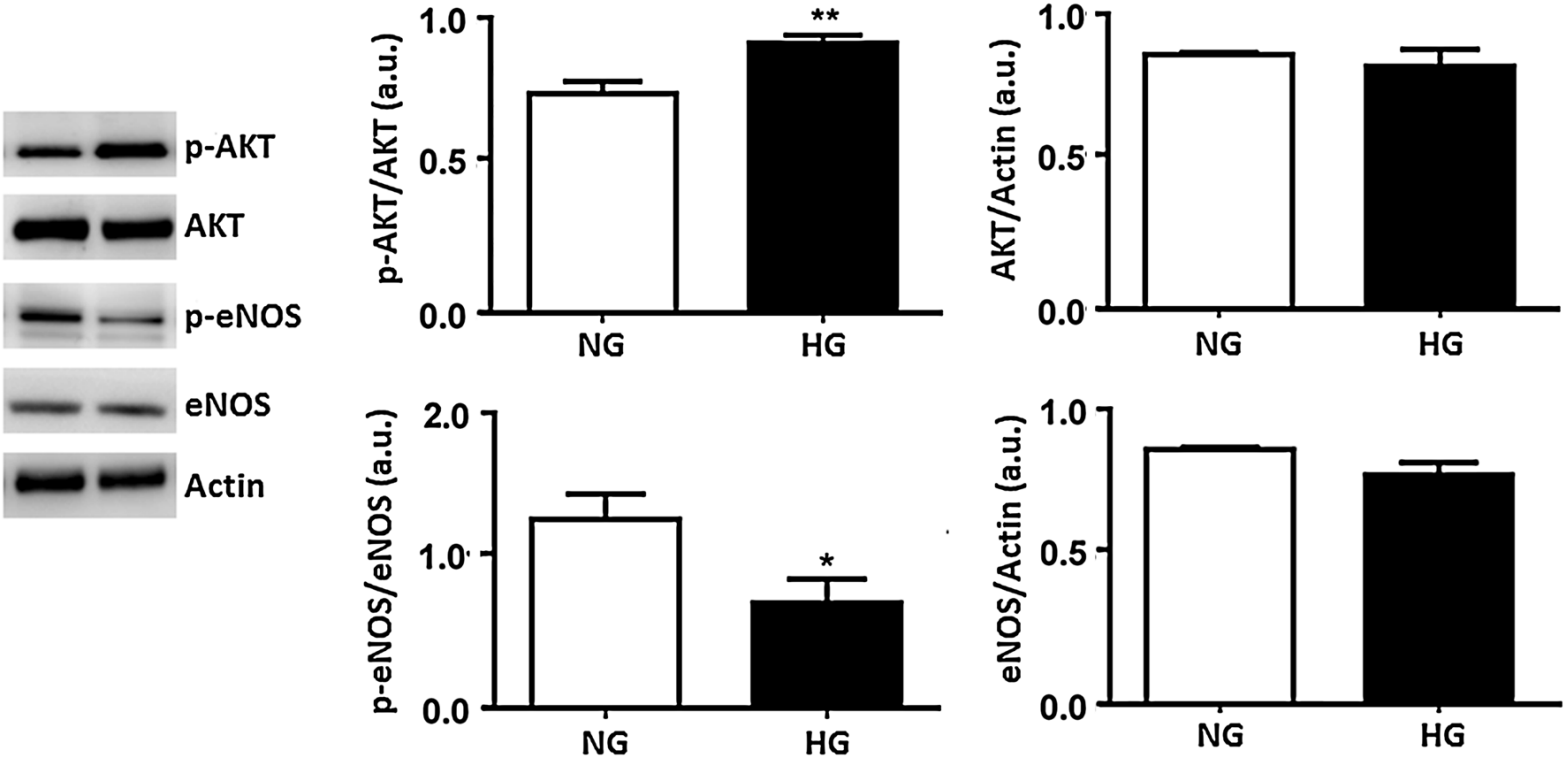
Administration of high glucose for 24 h enhanced phosphor-Ser473AKT and attenuated phospho-Ser1177eNOS in HUVECs. Whole cell lysates were prepared from confluent HUVECs exposed to 5 mmol/L (NG) or 25 mmol/L (HG) glucose for 24 h. AKT and eNOS phosphorylations were assayed for Western Blot analysis. The *panels* show representative images of different independent experiments. Densitometric values were normalized to total amounts of AKT and eNOS, respectively. *P < 0.05, **P < 0.01 HG vs. NG. *Bars* represent mean ± SEM for five independent experiments

It has been shown that impaired PI3K/AKT signaling due to hyperglycemia may promote endothelial dysfunction in diabetes [18]. To this aim, we firstly investigated the effects of acute HG in AKT phosphorylation and in its effector eNOS in HUVECs. In high glucose, phosphorylation levels of Ser473AKT increased, while phosphorylation levels of Ser1177eNOS reduced versus the control. The total amount of AKT and eNOS protein levels remained unchanged (Fig. 1). These results suggest that acute HG impairs AKT/eNOS signal transduction.

### Time course of insulin-mediated eNOS phosphorylation at Ser1177 in HUVECs

**Fig. 2:**
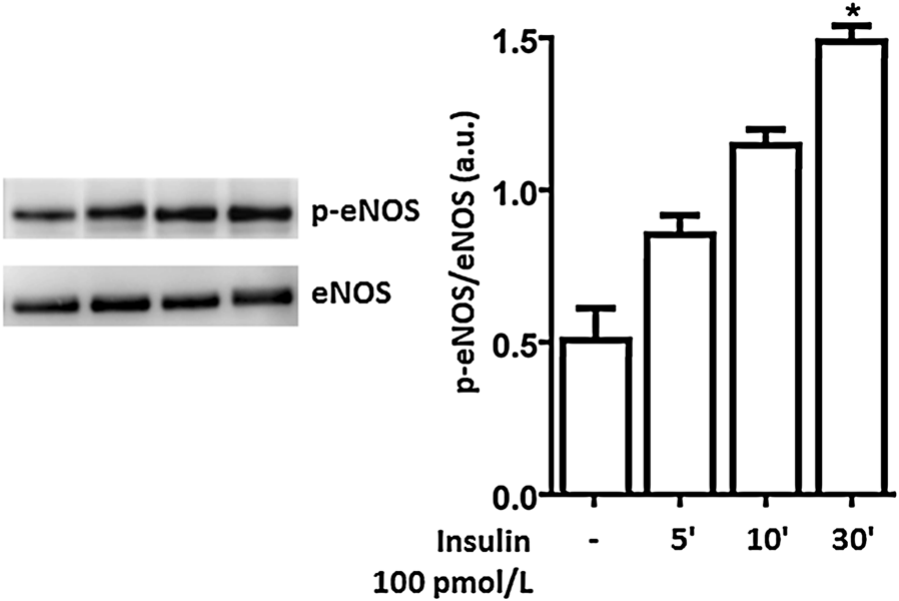
Insulin 100 pmol/L slightly increased phospho-Ser1177eNOS at 5 and 10 min, with its major effect occurring at 30 min. HUVECs were treated with 100 pmol/L insulin at different time points: 5, 10 and 30 min. Whole cell lysates were prepared for Western Blot analysis. The *panels* show representative images of different independent experiments. Densitometric values were normalized to total eNOS. *Bars* represent mean ± SEM for five independent experiments. *P < 0.05 NG with insulin 100 pmol/L vs. NG w/o insulin treatment

In order to establish the optimal amount of time for insulin incubation, confluent HUVECs were treated with 100 pmol/L insulin at three different time points: 5, 10 and 30 min. The insulin concentration used was 100 pmol/L insulin because it corresponds to physiological plasma insulin levels in humans, so this amount may be considered a good in vitro representation of in vivo insulin activity [16]. After insulin treatment, cells were collected and eNOS phosphorylation in Ser1177 levels were analyzed by Western Blot. We observed gradually increasing levels of Ser1177eNOS between 5 and 30 min, reaching statistical significance at 30 min (Fig. 2).

### Physiological insulin administration enhanced AKT and eNOS phosphorylation in NG in a concentration-dependent manner, but had no effects in HG in HUVECs

**Fig. 3:**
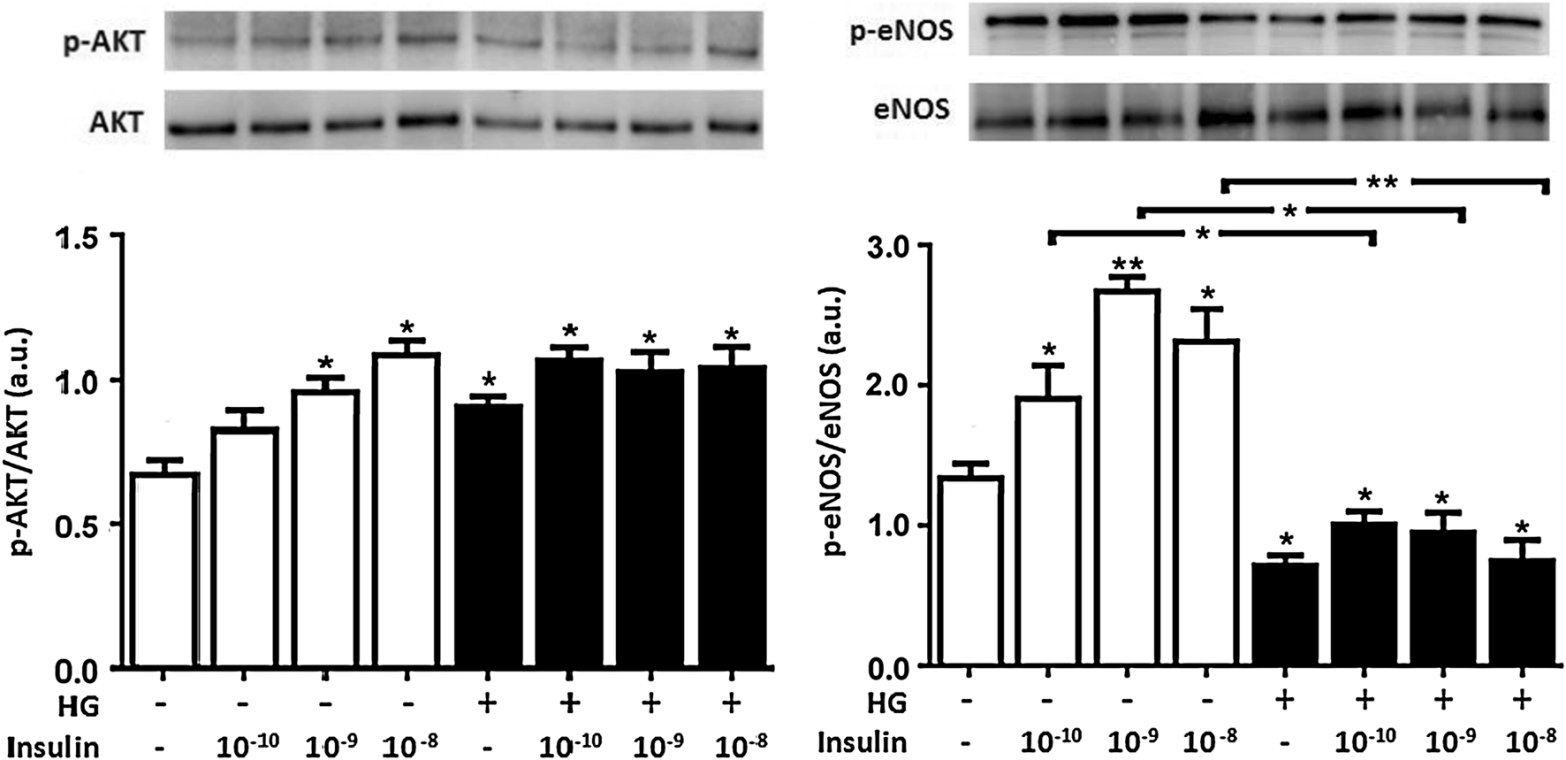
Insulin administration enhanced AKT and eNOS phosphorylation in NG in a concentration-dependent manner, but had no effects in HG in HUVECs. Confluent HUVECs were exposed to 5 mmol/L (NG) or 25 mmol/L (HG) glucose for 24 h. During the last 30 min HUVECs were treated with different physiological concentrations of insulin: 10^−10^ M = 100 pmol/L, 10^−9^ M = 10 nmol/L and 10^−8^ M = 1 nmol/L. Whole cell lysates were prepared for immunoblot analysis of AKT and eNOS phosphorylation. The *panels* show representative images of different independent experiments. Densitometric values were normalized to total amounts of AKT and eNOS, respectively. *Bars* represent mean ± SEM for five independent experiments. *P < 0.05, **P < 0.01 HG vs. NG

To investigate the alterations on the PI3K/AKT/eNOS-dependent insulin signaling pathway under HG conditions, activation of AKT and eNOS was determined by immunoblotting. Insulin administered at different physiological concentrations under NG conditions caused a concentration-dependent increase in phospho-Ser473AKT, which was statistically significant at the concentration of 10^−9^ and 10^−8^ M (Fig. 3). Contrary to what happened under NG, insulin physiological treatment under HG conditions did not cause the same effect.

To determine whether such a difference in endothelial AKT activity in response to insulin concentrations under the two conditions of NG and HG also had downstream consequences, we next evaluated the phosphorylation of eNOS. The different physiological insulin concentrations added at NG induced an increase in phospho-Ser1177eNOS that nicely correlated to a dose between 10^−10^ and 10^−9^ M, but was attenuated at 10^−8^ M. This could indicate a desensitization of the metabolic insulin signaling pathway. However, as the same insulin concentration continued to increase phosphorylated AKT levels, this indicated that insulin preserved some ability to signal to the metabolic arm.

Interestingly, physiological insulin added to HUVECs acutely treated under high glucose conditions did not follow the same eNOS phosphorylation profile that we observed in NG, indicating that insulin could not prevent high glucose-dependent inhibition of eNOS activation.

### Acute high glucose had no effects on ERK1/2, JNK and p38 phosphorylation levels in HUVECs, and physiological insulin treatment increased them under NG and HG conditions

**Fig. 4:**
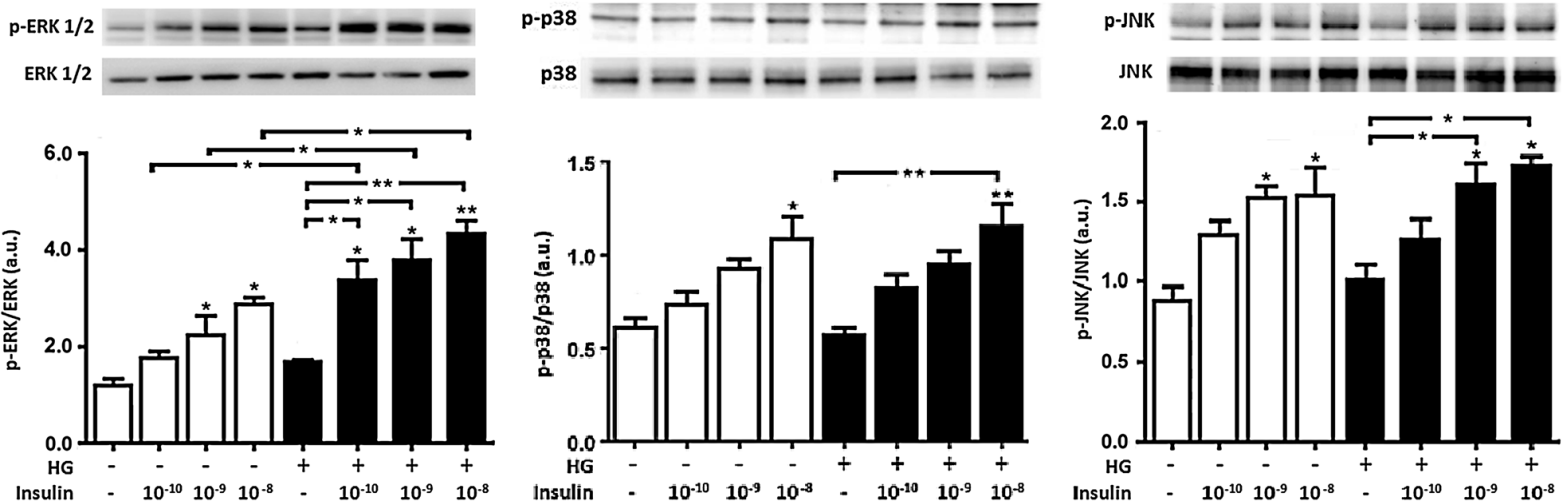
Administration of high glucose for 24 h had no effects on ERK1/2, p38 and JNK phosphorylations in HUVECs; however, insulin administration increased them under NG and HG conditions. Confluent HUVECs were exposed to 5 mmol/L (NG) or 25 mmol/L (HG) of glucose for 24 h. During the last 30 min, HUVECs were treated with different physiological concentrations of insulin, and whole cell lysates were prepared for Western Blot analysis. The *panels* show representative images of different independent experiments. Densitometric values were normalized to total amounts of ERK1/2, p38 and JNK, respectively. *Bars* represent mean ± SEM for five independent experiments. *P < 0.05, **P < 0.01 HG vs. NG

To investigate the alterations in the Ras/Raf/MAPK-dependent insulin signaling pathway in HG, activation of ERK1/2, p38 and JNK was determined. In NG, HUVECs responded to insulin stimulation with an increase in ERK1/2 phosphorylation in a concentration-dependent manner, being statistically significant for insulin doses 10^−9^ and 10^−8^ M. We obtained the same results for phospho-p38, which was statistically significant at insulin doses of 10^−8^ M, and phospho-JNK, with a significant increase at the insulin concentrations of 10^−9^ and 10^−8^ M (p < 0.05). Conversely to AKT and eNOS phosphorylation, 24 h of HG treatment did not affect the phosphorylated forms of ERK1/2, p38 or JNK. Intriguingly, insulin physiological treatment in HG maintained an increase of these forms in a concentration-dependent manner, and this increase was significant (1) at all insulin doses tested for phospho-ERK1/2, (2) at the concentration of 10^−8^ M for phospho-p38 and (3) at the concentrations of 10^−9^ and 10^−8^ M for phospho-JNK (Fig. 4).

### Acute high glucose attenuated IRβ expression in HUVECs. Insulin administration had no effects under NG and HG in terms of total amount but increased its phosphorylated form

**Fig. 5:**
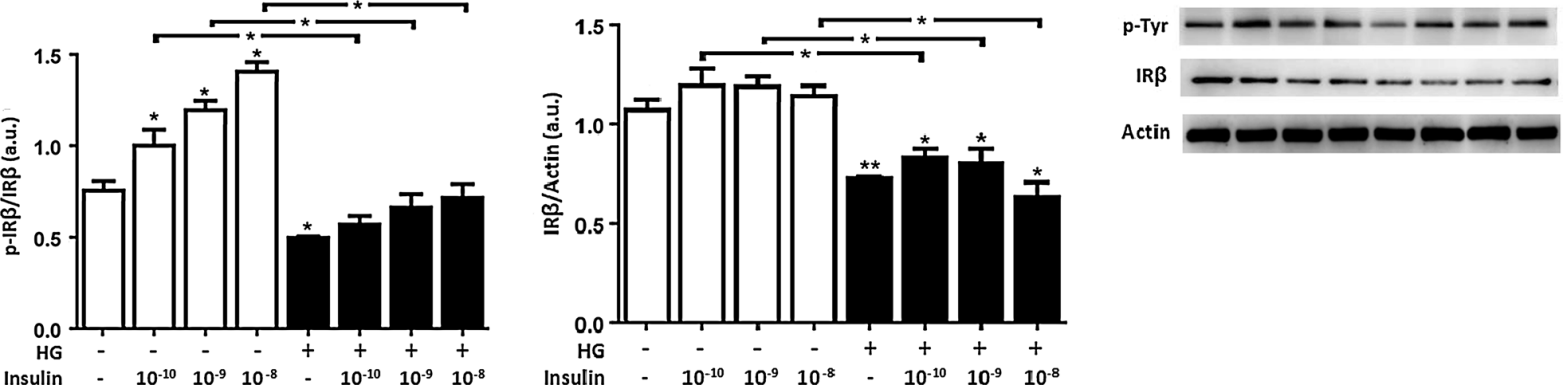
Administration of high glucose for 24 h attenuated IRβ total expression and its phosphorylated form in HUVECs. Physiological insulin increased IRβ tyrosine phosphorylation only for the condition of NG. Previously exposed to 24 h of 5 mmol/L (NG) or 25 mmol/L (HG) of glucose, cells were stimulated with different insulin concentrations for 30 min. Whole cell lysates were prepared for immunoblot analysis of the phosphorylated form of IRβ and the total amount of IRβ protein. The *panels* show representative images of different independent experiments. Densitometric values were normalized to total amounts of IRβ and actin, as indicated. *Bars* represent mean ± SEM for five independent experiments. *P < 0.05, **P < 0.01 HG vs. NG

We hypothesized that exposure of HUVECs to HG could affect the early steps of insulin signaling. To test this, IRβ protein expression levels were examined. Acute exposure of HUVECs to HG induced a significant decrease in IRβ tyrosine phosphorylation accompanied by a reduction in IRβ total protein versus the control (Fig. 5).

Insulin treatment under NG conditions enhanced in a dose-dependent manner IRβ tyrosine phosphorylation at all tested physiological insulin concentrations, but it had no effects under HG in HUVECs, although a statistically insignificant increase was observed. Insulin stimulation in NG had no effects on IRβ total expression compared with the untreated control. The decrease in IRβ total expression observed under HG conditions was not counteracted by any tested dose of insulin (Fig. 5).

## Discussion

In the present study, we show how HUVECs exposed to 24 h of high glucose lose the capacity to respond normally at physiological insulin concentrations. This phenomenon is only observed in the PI3K/AKT/eNOS pathway, while under the same conditions the Ras/Raf/MAPK pathway responds to insulin treatment with the up-regulation of phosphorylated forms of ERK1/2, p38 and JNK. This is consistent with the hypothesis that an imbalance occurs in the endothelial insulin signaling between pro- and anti-atherogenic arms with insulin preserving its ability to signal through the mitogenic arm, previously shown not to be affected under conditions of high insulin levels [19–21]. In a novel manner, we show that stimulation of endothelial insulin signaling with physiological insulin concentrations in HUVECs pre-exposed to 24 h high glucose: (1) on the one hand, is not sufficient to activate the PI3K/AKT/eNOS pathway, as has occurred in other studies involving lower insulin dose incubations [11, 22]; (2) and on the other, it does not affect the MAPK-mediated insulin pathway, which not only is maintained but also is exacerbated, and this occurs at very low insulin concentrations.

An altered PI3K/AKT/eNOS pathway is a common feature observed in cellular models exhibiting impaired endothelial function [5]. eNOS phosphorylation at Ser1177 is necessary for its maximal activation and, consequently, for optimal NO production [23]. In the present study, we show that in HUVECs expression of phospho-eNOS is decreased after 24 h of high glucose and that physiological insulin treatment under this condition cannot restore this activation.

PI3K and AKT are downstream effectors of insulin signaling and are also involved in other pathways implicated in proliferation and survival [24], apoptosis, angiogenesis and cellular transformation [17]. In our study, we observe that, when HUVECs are exposed to 24 h of high glucose, eNOS is not activated, while phospho-AKT is increased. These results suggest that under high glucose conditions AKT is probably directed to positively regulate proliferation and endothelial cell survival.

Attenuated insulin signaling has been proposed as resulting from alterations in both the IR and post-receptor signaling [25]. The IR is a heterodimeric protein complex, with an intracellular β-subunit (IRβ) and an extracellular α-subunit (IRα). Insulin binds to the IR through IRα, while the β subunit contains the intracellular tyrosin-kinase domain that initiates the signal cascade through the cytoplasm and the nucleus.

In the present study, we show that exposure to high glucose not only affects downstream IR signaling events, but also the total amount of intracellular β subunit. Under high glucose conditions, insulin is not able to phosphorylate the IRβ subunit, while it occurs under normal glucose conditions. Furthermore, IRβ total expression, which is decreased by high glucose, is unable to be restored through insulin treatment back to normal protein levels.

The decrease of IRβ total expression levels observed under high glucose potentiates the hypothesis that elevated AKT phosphorylation in this condition has consequences not only for eNOS activation, which directly depends on insulin signaling, but also for other effects governed by other pathways [17].

## Conclusions

Our study suggests that insulin at physiological concentrations is not preventive but may even contribute to the vascular impairment of endothelial phenotype previously damaged by high glucose, due to an increased pro-atherogenic pathway observed by an up-regulation of ERK1/2, p38 and JNK phosphorylation.

## Abbreviations

PI3K: phosphatidylinositol-4,5-bisphosphate 3-kinase
AKT: protein kinase B
eNOS: endothelial nitric oxide synthase
NO: nitric oxide
MAPK: mitogen-activated protein kinase
ERK1/2: extracellular signal-regulated kinase 1/2
JNK: jun-amino(N)-terminal kinase
ET-1: endothelin-1
HUVECs: human vein endothelial cells
NG: normal glucose
HG: high glucose
Ins: insulin
IR-α/β: insulin receptor-alpha/beta subunit
IRS-1/2: insulin receptor substrates-1/2
NG: normal glucose
HCAECs: human coronary artery endothelial cells
